# The Impact of ZIF-8 Particle Size Control on Low-Humidity Sensor Performance

**DOI:** 10.3390/nano14030284

**Published:** 2024-01-30

**Authors:** Sang Jun Kim, Jaemin Lee, Jong-Seong Bae, Jung Woo Lee

**Affiliations:** 1 Institute of Materials Technology, Pusan National University, Busan 46241, Republic of Korea; ksj0125@pusan.ac.kr; 2 Department of Materials Science and Engineering, Pusan National University, Busan 46241, Republic of Korea; zld0315@pusan.ac.kr; 3 Busan Center, Korea Basic Science Institute, Busan 46742, Republic of Korea; jsbae@kbsi.re.kr

**Keywords:** humidity sensor, MOF, ZIF-8, particle size, capacitive sensor

## Abstract

An accurate humidity measurement is essential in various industries, including product stability, pharmaceutical and food preservation, environmental control, and precise humidity management in experiments and industrial processes. Crafting effective humidity sensors through precise material selection is crucial for detecting minute humidity levels across various fields, ultimately enhancing productivity and maintaining product quality. Metal–organic frameworks (MOFs), particularly zeolitic imidazolate frameworks (ZIFs), exhibit remarkable properties and offer a wide range of applications in catalysis, sensing, and gas storage due to their structural stability, which resembles zeolites. The previous research on MOF-based humidity sensors have primarily used electrical resistance-based methods. Recently, however, interest has shifted to capacitive-based sensors using MOFs due to the need for humidity sensors at low humidity and the resulting high sensitivity. Nevertheless, further studies are required to optimize particle structure and size. This study analyzes ZIF-8, a stable MOF synthesized in varying particle sizes, to evaluate its performance as a humidity sensor. The structural, chemical, and sensing properties of synthesized ZIF-8 particles ranging from 50 to 200 nanometers were examined through electron microscopy, spectroscopic, and electrochemical analyses. The fabricated copper electrodes combined with these particles demonstrated stable and linear humidity sensing capabilities within the range of 3% to 30% relative humidity (RH).

## 1. Introduction

Humidity measurement plays a crucial role in various fields [1]. It is needed to maintain product stability [2], preserve pharmaceuticals and food items [3], understand the impact of indoor humidity on human health [4], and to achieve precise humidity control in experiments and industrial processes [5]. In areas such as battery and semiconductor production, there is a high demand for accurate sensing at minute humidity levels, especially in low-humidity areas [5]. Therefore, fabricating humidity sensors through the appropriate material selection holds significant importance.

Recent research has been actively conducted on humidity sensors utilizing various materials such as metal oxides [6], composites [7], 2D materials [8], and carbon-based materials [9]. Especially, humidity sensors using carbon ink operate reliably over a wide range and attract significant attention, functioning even in conditions below 10% RH [10]. However, for industrial applications, there is a need for research on sensors specialized in low humidity with enhanced sensitivity [5]. To enhance the overall sensitivity of sensors, the most effective strategy involves improving adsorption performance by maximizing the surface area [11]. Therefore, there is a need for research on porous materials with significantly larger surface areas compared to conventional sensor materials. Metal–organic frameworks (MOFs) represent porous materials that allow for easy synthesis of diverse structures and properties via various combinations of metals and linkers [12]. MOFs have a wide range of applications, including catalysis [13], sensors [14], and gas storage [15]. The zeolitic imidazolate framework (ZIF) is a type of MOF composed of linkers like imidazole and transition metal ions, like zinc or cobalt [16]. The ZIF exhibits an arrangement where organic linkers and transition metal ions are combined in a zeolite-like structure, offering higher structural stability compared to conventional MOFs [17]. Hence, diverse applications, such as physical ion adsorption or chemical oxidation/reduction reactions, are feasible [18].

Humidity sensors are designed based on various types, including resistance, capacitance, conductance, and electrochemical sensors. Despite the ongoing research on humidity sensors utilizing MOFs, a predominant focus has been on resistance-based sensors in most studies [19,20]. Resistance-based sensors, although advantageous for mass production due to their simple structure, face difficulty in measuring at low-humidity levels due to significant increases in resistance [1]. On the other hand, capacitance-based humidity sensors, while capable of measuring low-humidity ranges, incur higher production costs, owing to their more complex structure [1]. Recently, attention has been drawn to the research utilizing MOFs for simple structured capacitance-based sensor fabrication for humidity measurement [21,22,23]. However, there is a lack of research regarding optimization concerning particle structure or size, indicating a need for further exploration.

This study aims to synthesize ZIF-8, a stable MOF, in various particle sizes and analyze its humidity sensor performance. The synthesized ZIF-8 was subjected to various electron microscopy and spectroscopy analyses to evaluate its structural and chemical characteristics. Additionally, patterned copper electrodes were fabricated to measure the sensor’s performance. The humidity sensor performance was evaluated using the capacitance measurement method, employing an LCR meter to ascertain stable humidity sensing capabilities within the range of 3% to 30% RH.

## 2. Materials and Methods

### 2.1. Synthesis of ZIF-8

The synthesis process of ZIF-8 was conducted by referring to existing the literature [24,25]. Initially, 1.29 g of zinc nitrate hexahydrate (Zn(NO_3_)_2_∙6H_2_O, Sigma Aldrich, St, Louis, MA, USA, 98%) was dissolved in 100 mL of methanol (CH_3_OH, Honeywell, Charlotte, NC, USA, 99.9%) to synthesize 50 nm particles. Simultaneously, another 100 mL of methanol was used to dissolve 1.32 g of 2-methylimidazole (Sigma Aldrich, St, Louis, MA, USA, 99%) to create a transparent solution. The two solutions were mixed and vigorously stirred for about 5 min to ensure thorough mixing. After stirring, the mixture was left undisturbed at room temperature for approximately 24 h for synthesis. After a white solution formed, the product was washed three times with methanol at 7000 rpm for 10 min each in a centrifuge. The resulting material was then dried in a vacuum oven at 60 °C for at least 12 h to obtain white powder. The 200 nm particles were synthesized from the 50 nm particles by replacing the zinc precursor with 0.88 g of zinc acetate dihydrate (Zn(CH_3_COO)_2_∙2H_2_O, Junsei, Tokyo, Japan, 99%), using the same procedure. The 100 nm particles were synthesized using a slightly different method. Initially, 1.49 g of zinc nitrate and 3.30 g of 2-methylimidazole were dissolved separately in 56 mL of methanol, each. The fully dissolved 2-methylimidazole solution was then added dropwise to the zinc nitrate solution using a pipette. The resulting mixture was stirred at room temperature for 1 h using a magnetic stirrer, and then subjected to the same process as described earlier using a centrifuge and vacuum oven to obtain white powder.

### 2.2. Fabrication of Copper Electrode

**Figure 1:**
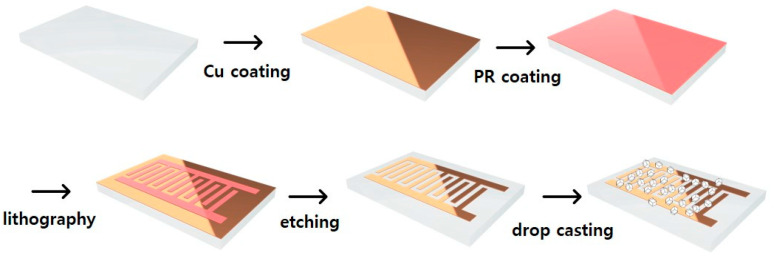
Schematic of humidity sensor fabrication using ZIF-8 on the interdigit-patterned Cu electrode/glass substrate.

The fabrication process of the copper electrode was schematically depicted in Figure 1. Initially, a copper film of 200 nm thickness was deposited on a glass substrate using an electron beam evaporator. Subsequently, to pattern the deposited copper film as desired, photolithography technology was employed. Positive photoresist (PR, AZ 5214-E, AZ Electronic Materials, NJ, USA) was spin-coated onto the glass substrate with the deposited copper film at 3000 rpm and thermally treated at approximately 95 °C for 1 min. Following this, exposure was carried out using a mask (Figure S1) designed with an interdigital pattern and a mask aligner (MDA 400S, MIDAS SYSTEM, Daejeon, Korea), followed by the development and etching of the deposited copper film. Finally, residual photoresist was washed away to reveal the pattern.

### 2.3. Fabrication Method of Humidity Sensor

100 mg of synthesized ZIF-8 was dispersed in 500 μL of ethanol (C_2_H_5_OH, Daejung, Siheung, Korea, 95%) using an ultrasonic sonicator for 10 min. The prepared copper electrode was placed on a hotplate at 70 °C, and the dispersed solution was dropped onto the electrode area using a pipette. After approximately 10 min of evaporating the ethanol on the hotplate, the material was dried in a vacuum oven at 100 °C for over 12 h to completely remove moisture. Extension wires were created using copper tape and conductive paste (silver paste, Elcoat, CANS, Tokyo, Japan) to connect the electrode.

### 2.4. Structural Analysis of ZIF-8

The particle morphology analysis of the synthesized ZIF-8 was conducted using field-emission scanning electron microscopy (FE-SEM, Mira 3, Tescan, Brno, Czech Republic) equipped with an energy-dispersive spectroscopy (EDS, Super X, FEI company, Hillsboro, OR, USA), as well as a field emission transmission electron microscopy (FE-TEM, Talos F200X, Thermo Fisher Scientific, MA, USA). Additionally, the crystallographic and chemical bonding characteristics were analyzed using X-ray diffraction analysis (XRD, ULTIMA IV, Rigaku, Tokyo, Japan) and a monochromatic X-ray photoelectron spectrometer (XPS, K-Alpha+, Thermo Fisher Scientific, MA, USA) with Al Kα (1486.6 eV). The XPS peak was deconvoluted using XPSpeak41.

### 2.5. Humidity Measurement Method

An airtight acrylic chamber was constructed with an onboard humidity sensor (THD-DD1, autonics, Busan, Korea) to enable humidity measurement inside. Inlet and outlet ports for dry gases were created, allowing the control of gas injection and evacuation. An internal fan was installed for rapid gas mixing. Nitrogen gas (N_2_, 99.9%) was slowly injected to gradually reduce the internal humidity to the desired level. Once stabilized, the humidity measurement commenced. The capacitance was measured with an Agilent E4980A LCR meter in Cp-D mode. The measurements were conducted within a stable frequency range of 1 to 1000 kHz. 

## 3. Results

**Figure 2:**
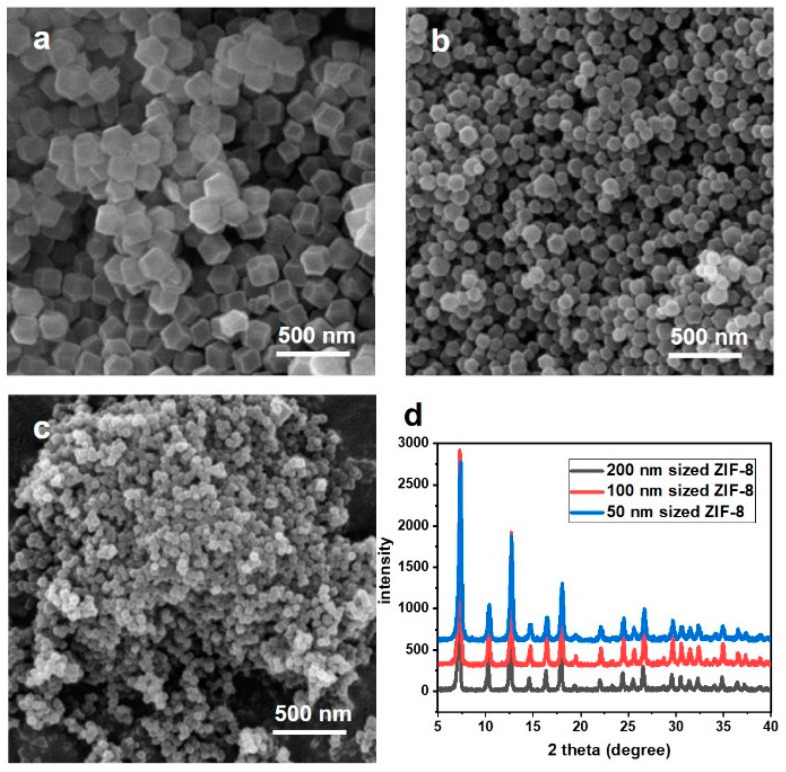
(**a**–**c**) SEM images and (**d**) XRD graphs of ZIF-8 at different sizes.

SEM analysis was conducted to confirm the size and shape of the synthesized ZIF-8, and the results are depicted in Figure 2. ZIF-8 of various sizes were synthesized according to the methods described in Section 2.1. Figure 2a shows an image of a ZIF-8 with a size of 200 nm. As observed in the image, ZIF-8 exhibits a rhombic dodecahedron shape [26]. This shape constitutes a polyhedron formed by 12 rhombic faces. The particle sizes are uniformly distributed overall, and most of the particles exhibit distinct shapes. Figure 2b illustrates the shape of the particles when they were formed by adding one drop at a time using a syringe. Although the particle shape in Figure 2a and the rhombic dodecahedron are identical, the particle size appears to be reduced to approximately 100 nm. Moreover, when changing the zinc precursor from zinc nitrate to yield the precursor, as shown in Figure 2c, smaller particles of about 50 nm were created with the same shape. Hence, it is evident that controlling factors, such as the type of zinc precursor or the coupling rate of metal and linker, enable the size modulation of particles.

The crystallinity of ZIF-8 can be identified through XRD analysis. Figure 2d represents the XRD patterns of the synthesized ZIF-8 with various particle sizes. The distinct peaks at consistent positions for each particle confirm the identical crystallinity of the substance. A closer inspection of the peaks in the graph confirms clear peaks at 7.3°, 10.3°, 12.7°, 14.8°, 16.4°, and 18.0°, corresponding to the (110), (200), (211), (220), (310), and (222) planes, respectively [27]. Particularly, the ZIF-8, being a porous material, demonstrates a significant main peak at below 10°, indicating its particle structure with nano-sized pores. This aligns precisely with the theoretically simulated XRD peaks (JCPDS no.62-1030) calculated based on the structure of ZIF-8 [28].

**Figure 3:**
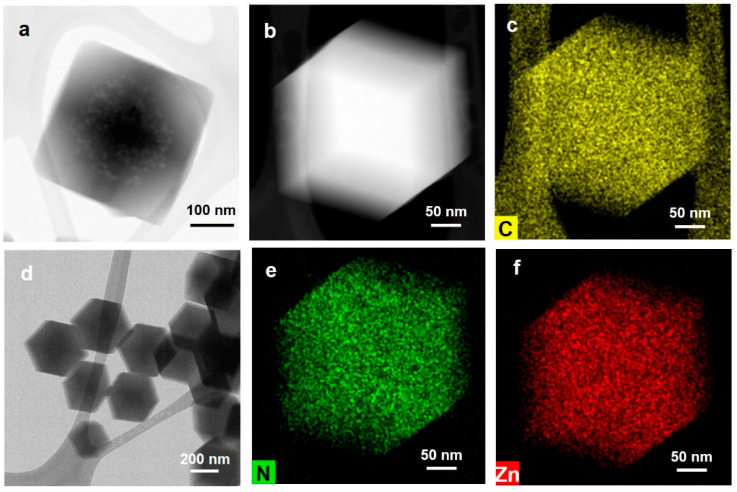
Structural analysis of ZIF-8 by (**a**,**d**) TEM, (**b**) STEM, (**c**,**e**,**f**) EDX analysis.

We conducted a HR-TEM analysis to delve deeper into the structure of the particles. Particles with a rhombic dodecahedron shape exhibit square or hexagonal cross-sections depending on the direction, as seen in Figure 3a,b. Figure 3d confirms the uniform synthesis of particles with distinctly defined boundaries when observing multiple particles. Figure 3c,e,f illustrate the elemental distribution through EDX analysis in Figure 3b. Carbon, nitrogen, and zinc are uniformly distributed across the particles, indicating the uniform synthesis of ZIF-8. Thus, TEM, SEM, and XRD collectively verify the uniform synthesis of highly crystalline ZIF-8 particles at sizes of 50 nm, 100 nm, and 200 nm.

**Figure 4:**
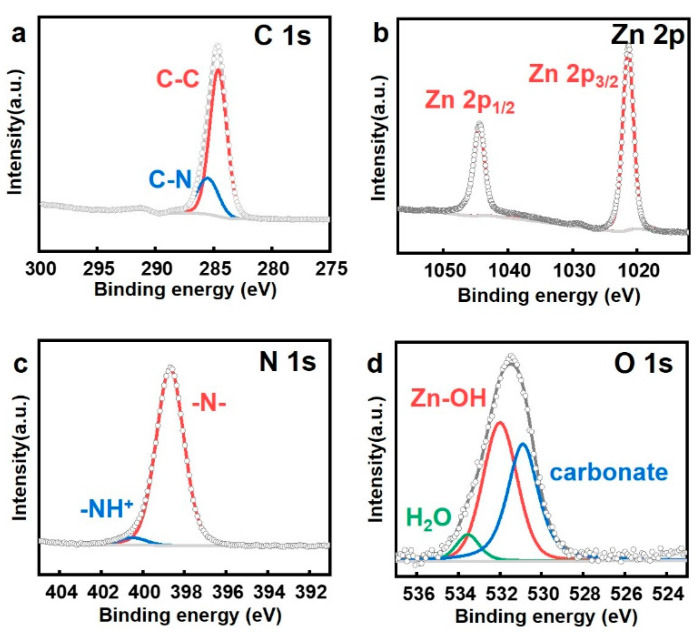
Analysis of the (**a**) C 1s, (**b**) Zn 2p, (**c**) N 1s, and (**d**) O 1s bonds in ZIF-8 by XPS analysis.

The chemical bonding in the synthesized ZIF-8 was examined using XPS analysis, as depicted in Figure 4. Figure 4a presents the C 1s peak graph, where the peaks at 284.6 eV and 285.5 eV represent C-C and C-N bonds, respectively [29]. Figure 4b displays the Zn 2p graph, indicating oxidation states within ZIF-8, with peaks at 1021.4 eV and 1044.4 eV, corresponding to the binding energies of Zn^2+^’s 2p_3/2_ and 2p_1/2_, respectively [30]. This confirms the existence of Zn in ZIF-8 as Zn^2+^. The N 1s graph reveals two peaks: one at 398.6 eV, representing pyrrolic N, mainly appearing in nitrogen peaks of ZIF-8 crystals, and the other at 400.5 eV, indicating pyridinic N, found in the boundaries or defects of the structure, bonded to only one carbon [24]. The oxygen peak (O 1s) does not inherently exist in ZIF-8; however, it appears due to various surface-active substances, such as H_2_O, -OH, and carbonate from the atmosphere, which can bond to the surface of ZIF-8 [31].

The SEM image of the humidity sensor electrode, fabricated by drop casting on a Cu electrode produced using lithography, is presented in Figure S2. To facilitate a comparison with the electrode, uncoated regions were intentionally created and observed in the top view, as depicted in Figure S2a,b. The white regions represent ZIF-8, clearly distinguished from the black Cu electrode. It is evident that ZIF-8 is densely distributed across the electrode surface. Figure S2c illustrates a side view of the fabricated humidity sensor, while Figure S2d shows a side view of the Cu electrode alone. Through these images, it is discernible that the thickness of the Cu electrode is approximately 100–200 nm, with a subsequent deposition of ZIF-8 extending up to around 1 micrometer. This observation confirms the formation of the humidity sensor, wherein the ZIF-8 layer overlays the Cu electrode.

**Figure 5:**
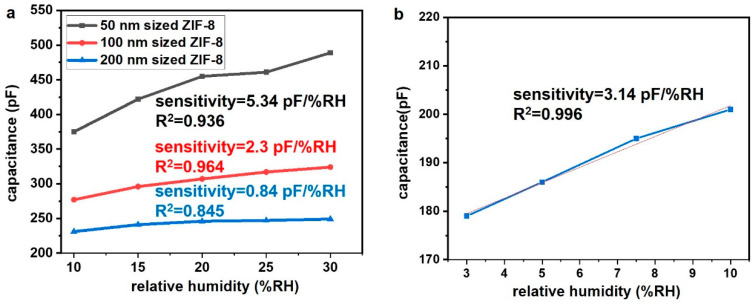
(**a**) Graphs of capacitive humidity measurements using different sizes of ZIF-8 under 500 Hz, (**b**) Graphs of capacitive humidity measurements in a low-humidity area using ZIF-8 with a particle size of 200 nm under 100Hz.

Figure 5 shows graphs of the capacitance-based humidity measured via the LCR meter using electrodes prepared with the synthesized ZIF-8 of various sizes. A capacitance-type humidity sensor typically exhibits a tendency where capacitance and sensitivity decrease as the frequency increases [32]. However, finding a frequency with good linearity is more crucial than achieving high sensitivity. Figure 5a illustrates the measurement results at 500 Hz, which demonstrates the highest linearity among various frequencies. Electrodes made with different sizes of ZIF-8 exhibit linear sensor performance within the range of 10–30% RH. The electrode utilizing 200 nm-sized ZIF-8 demonstrates a linear humidity sensor with 0.84 pF/% RH and R^2^ = 0.845. The sensor employing 100 nm ZIF-8, featuring a smaller particle size, displays increased sensitivity and linearity with 2.30 pF/% RH and R^2^ = 0.964, respectively. The electrode prepared with the smallest 50 nm ZIF-8 shows the highest sensitivity at 5.34 pF/% RH and R^2^ = 0.936, exhibiting excellent linearity. The results indicate that as particle size decreases, higher sensitivity is achieved. This is attributed to changes occurring due to water molecules adhering to the ZIF-8 surface, affecting the capacitance. Smaller particles have a larger surface area, where adsorbed particles directly contact the MOF, leading to a more significant change in capacitance [33]. Additionally, the electrode utilizing 200 nm ZIF-8 exhibits linearity within the range of 3–10% RH, indicating its potential usability as a humidity sensor, even in low-humidity conditions.

## 4. Conclusions

This study demonstrated the influence of particle size variation in humidity sensor performance using synthesized ZIF-8 of different sizes. SEM and HR-TEM analyses confirmed that particles from 50 nm to 200 nm maintained similar shapes while being size-adjusted. Smaller particles exhibited higher sensitivity and linear humidity changes due to a larger surface area interacting with water molecules, causing significant changes in capacitance. The crystallinity and chemical properties of ZIF-8 were verified through XRD and XPS analyses. These results suggest the technological advancement potential for precise humidity control and measurement, catering to future subtle environmental changes and industrial demands using humidity sensors, based on the various sizes of ZIF-8.

## Data Availability

Data are contained within the article.

## Supplementary Materials

The following supporting information can be downloaded at: https://www.mdpi.com/article/10.3390/nano14030284/s1, Figure S1. Drawing diagram for manufacturing Cu electrodes.; Figure S2. SEM images of the top (a,b) and side (c) views of ZIF-8 deposited on a Cu electrode. (d) SEM image of side view of Cu electrode only; Figure S3. Graph of capacitive humidity measurements in the range 10-50 %RH using various sizes of ZIF-8.; Figure S4. Response and recovery times of 50 nm sized ZIF-8 ca-pacitive humidity sensor.
