# Supplementary material for: The Impact of ZIF-8 Particle Size Control on Low-Humidity Sensor Performance
Source: Nanomaterials (Basel). 2024 Jan 30;14(3):284. doi: 10.3390/nano14030284 (PMC10857053; doi:10.3390/nano14030284)
Supplement: Supplementary file 1 [file nanomaterials-14-00284-s001.zip › nanomaterials-2833412-supplementary.pdf]

Supporting Information

The Impact of ZIF-8 Particle Size Control on Low Humidity Sensor Performance

Sang Jun Kim [1], Jaemin Lee [2], Jong-Seong Bae [3] and Jung Woo Lee [2,*]

[1] Institute of Materials Technology, Pusan National University, Busan 46241, Republic of Korea; ksj0125@pusan.ac.kr
[2] Department of Materials Science and Engineering, Pusan National University, Busan 46241, Republic of Korea; zld0315@pusan.ac.kr
[3] Busan Center, Korea Basic Science Institute, Busan 46742, Republic of Korea; jsbae@kbsi.re.kr
* Correspondence: jungwoolee@pusan.ac.kr

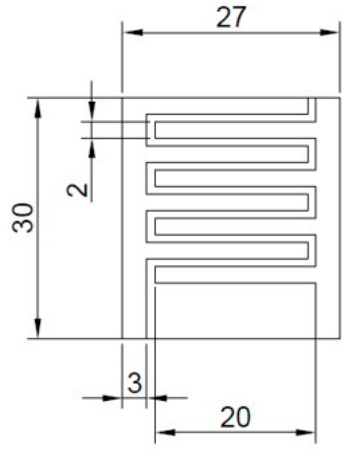

Figure S1. Drawing diagram for manufacturing Cu electrodes.

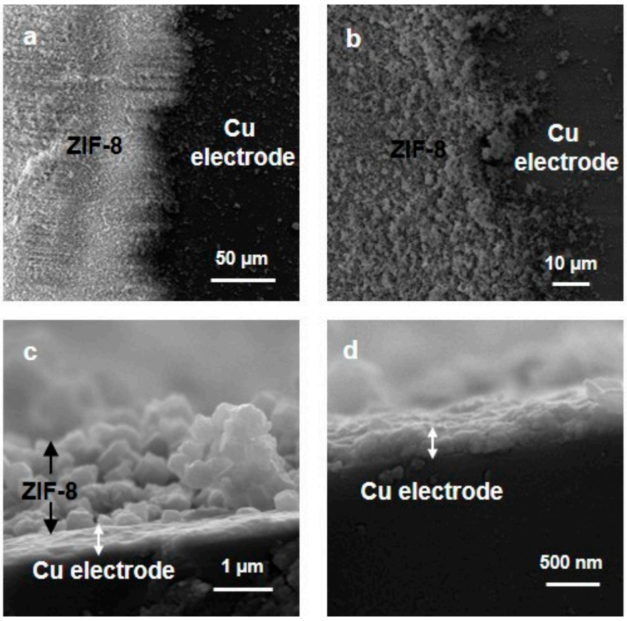

Figure S2. SEM images of the top (a,b) and side (c) views of ZIF-8 deposited on a Cu electrode. (d) SEM image of side view of Cu electrode only.

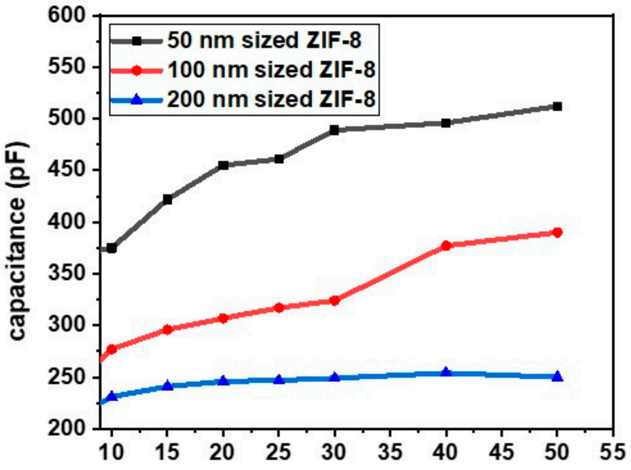

Figure S3. Graph of capacitive humidity measurements in the range 10-50 %RH using various sizes of ZIF-8.

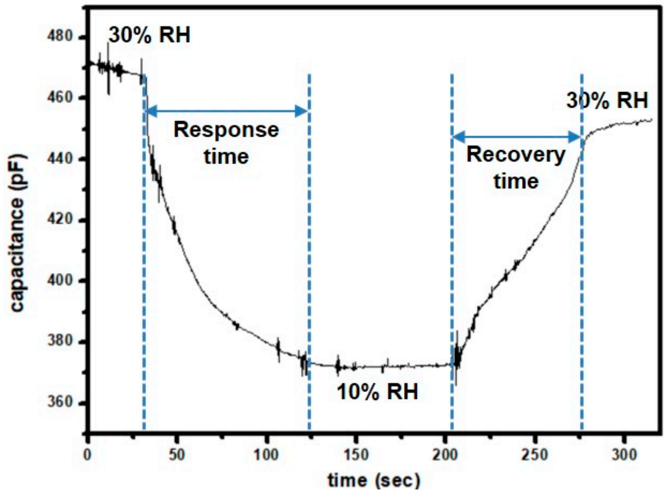

Figure S4. Response and recovery times of 50 nm sized ZIF-8 capacitive humidity sensor.
